# Astrocytic-Neuronal Teamwork Against External Iron Attacks: Does It Always Work?

**DOI:** 10.1093/function/zqab009

**Published:** 2021-02-22

**Authors:** Seojin Lee, Gabor G Kovacs

**Affiliations:** 1 Department of Laboratory Medicine and Pathobiology, Tanz Centre for Research in Neurodegenerative Disease, University of Toronto, Toronto, ON, Canada; 2 Laboratory Medicine Program & Krembil Brain Institute, University Health Network, Toronto, ON, Canada

## A Perspective on “Iatrogenic Iron Promotes Neurodegeneration and Activates Self-protection of Neural Cells against Exogenous Iron Attacks”

In this issue of the Journal, Xia et al. address the question of the possible role of iatrogenic iron from metal implants on the development of neurological diseases.^1^ Iron is the brain’s destined partner; it is essential for the physiological function but slight fluctuations in its levels deem detrimental to neuronal health. There is growing understanding of the tightly regulated mechanism of brain iron homeostasis, which involves diverse proteins across different cellular populations mediating two species of iron present in the central nervous system (CNS): the ferric (Fe^3+^) and ferrous (Fe^2+^) iron. The latter is particularly thought to produce reactive oxygen species to elicit neurotoxicity under excessive levels.^2^

Eventually with aging, iron accumulates in the human brain, exhibiting an anatomical selective vulnerability demonstrated by a pronounced accumulation, particularly in the basal ganglia.^3^ Intensified iron deposition is demonstrated in many neurodegenerative conditions characterized by progressive nerve cell dysfunction and accumulation of pathologically altered proteins, suggesting a link between their pathological process and the excess brain iron. Indeed, iron deposition in these conditions is focused in brain regions underlying the main disease pathologies such as the substantia nigra of Parkinson’s disease (PD) brains, the striatum in multiple system atrophy (MSA), globus pallidus in progressive supranuclear palsy (PSP), and the frontal cortex and hippocampus of Alzheimer’s disease (AD) brains.^4^^,^^5^ Further studies suggest a mutual and pathogenic interaction between iron and the neurodegeneration-related proteins.^6^

This study^1^ further elaborates on this delicate partnership, focusing specifically on PD. Examining the relationship between metal implantation and occurrence of PD as well as other cerebral diseases by retrospective analysis of patient data, their study revealed a greater number of metal transplant history in ischemic stroke and PD patients compared to healthy controls and a higher occurrence of PD in patients who underwent orthopedic surgeries with metal implants compared to those performed without. In addition, the study demonstrated increased serum iron, ferritin, and transferrin (Tf) concentrations in patients with metal implants supporting their concept that metal implantation results in iron homeostasis imbalance.

Investigating the underlying mechanism of exogenous iron effects on neuronal function, mice hypodermically injected with iron dextran were examined and the study demonstrated increased serum iron levels and brain iron deposition. Astrocytic and microglial up-regulation of messenger ribonucleic acid (mRNA) and protein expression of a major cellular iron import protein, divalent metal transporter 1 (DMT1), was demonstrated with a contrasting down-regulation in neurons which showed increased mRNA levels of Nedd4 family interacting protein (Ndfip1), a key player in DMT1 degradation. Furthermore, the study demonstrated reactive astrogliosis, microglial activation, and apoptosis in mouse brains and behavioral changes in mice in response to the iron dextran treatment. Based on these findings, the study concludes that excess iron, including that from surgical implants in humans, should be considered a risk factor for neurodegenerative disorders (NDDs).

Iron involvement in the pathogenesis of NDDs is increasingly recognized by the emerging studies demonstrating the direct interaction between iron and the disease-hallmark proteins including alpha-synuclein in PD. Fundamentally, iron and alpha-synuclein are seemed to be mutually involved in their physiological and pathological cellular homeostasis.^6^ However, given the bi-directionality of their interaction and the fundamental limitation in studies to delineate this phenomenon, such as end-stage disease state of post-mortem samples, the primary question is which comes first—iron accumulation or protein pathology? This article uniquely explores the possibility of the former scenario first by using a retrospective analysis approach of current PD patients, supporting the viewpoint that iron burden precedes disease pathology. The article also describes the emergence of neuroprotective mechanisms as a cellular response to excess iron, emphasizing that glial cells present as key players in the process, supporting their importance in neurodegeneration which was underappreciated for long. Findings of this article,^1^ including the involvement of Ndfip4 underlying neuroprotection, reinforces previous studies showing regional expression changes of different iron homeostatic proteins in NDDs,^7^^,^^8^ which together with the differential involvement of the proteins across different cell types, also pointed to an increased iron uptake by glia.

With a combination of clinical patient data analysis and experiments *in vivo*, the authors suggest the consideration of surgical metal implants as a risk factor for NDDs. Establishment of the suggested link merits future studies with a wider selection of disease subjects, including patients of other iron-associated NDDs such as AD, PSP, and MSA. Demonstration of protein pathology, or at least dysfunction of cellular protein processing systems characteristic of PD in the mice models could further provide a significant support of the proposed risk for NDDs.

**Figure 1.**
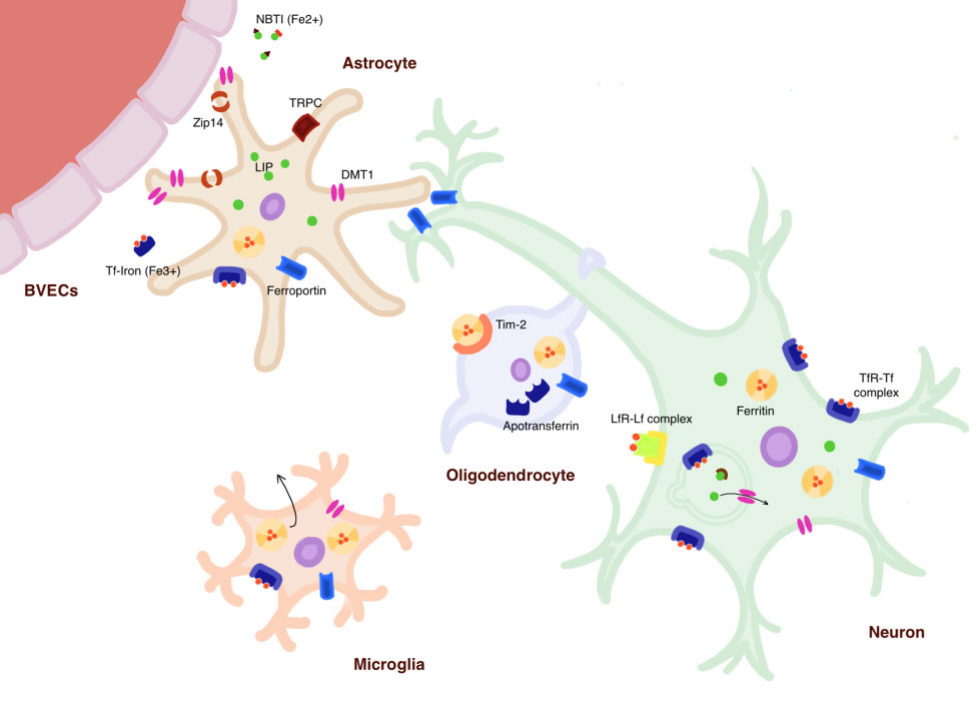
Iron Homeostasis in the CNS. Iron enters the CNS through the blood–brain barrier made up of brain vascular endothelial cells. In the brain interstitial fluid (ISF), it exists in two forms: ferrous iron (Fe^2+^) bound to ATP or citrate called non-Tf bound iron and ferric iron (Fe^3+^) bound to Tf (holo-Tf). Iron enters astrocytes mostly by DMT1s, but also by metal transporter ZIP14 and transient receptor potential canonical channels. Iron is stored in cells either as pool of ferrous iron referred to as liable iron pool or as ferric iron bound to ferritin, the major cellular iron storage protein. In neurons, iron enters mostly through the Tf cycle, which refers to the formation of clathrin-coated pit upon binding of holo-Tf to Tf receptor (TfR) on the membrane, then formation of early endosome which iron is released from Tf by endosomal proton efflux and exit into the cytoplasm through DMT1s. Iron can also enter neurons through DMT1s and lactoferrin (Lf) receptors which ferric iron-bound Lf binds for internalization. In oligodendrocytes, ferritin can be taken up through the membranous protein, t-cell immunoglobin, and mucine domain-containing protein-2, and it is responsible for the synthesis and release of Tf proteins. In microglia, iron enters the cell through DMT1s and TfRs, and it harbors mechanism to release ferritin into the CNS. Universally across the cell types, iron is exported out into the ISF by a membranous protein, ferroportin. Modified summary of Xu et al. and Nnah and Wessling-Resnick.^9^^,^^10^

Let us step back and get the bird’s eye view on the findings of this study. Despite the demonstrated mechanism of neuroprotection (1) apoptosis is still prevalent in iron-treated mouse brains; and (2) PD occurrence still seems to be higher in individuals with histories of metal implantations. This discrepancy suggests that the iron-induced neuroprotective mechanisms might be limited or unsuccessful. To tackle this question, the ultimate fate of iron-packed glia and their effects on the CNS, and the cellular expression changes of other iron homeostatic proteins in response to elevated iron levels need to be further investigated. Iron homeostasis is mediated by a number of import, storage, and export proteins in each cell type^9^ (Figure 1). Comprehensive understanding of the cellular response to abnormal iron levels will require co-examination of these proteins.

In summary, this article addresses a critical relationship of external iron to NDD. Of particular interest is whether exogenous iron from a yet underestimated external source can act as a deal-breaker driving the brain into neurodegeneration, or the brain is able to mobilize a successful protective mechanism. The study by Xia et al.^1^ provides a detailed examination of the brain response to external iron, instigating further research examining iron homeostasis by distinct cell types and neuroprotective changes in pathological conditions.

## Funding

This work was supported by the Rossy Foundation.

## Conflict of Interest Statement

The authors declare no conflict of interest.
